# Progress Toward Containment of Poliovirus Type 2 — Worldwide, 2017

**DOI:** 10.15585/mmwr.mm6624a5

**Published:** 2017-06-23

**Authors:** Nicoletta Previsani, Harpal Singh, Jeanette St. Pierre, Liliane Boualam, Jacqueline Fournier-Caruana, Roland W. Sutter, Michel Zaffran, MEng

**Affiliations:** ^1^World Health Organization, Geneva, Switzerland; ^2^Division of Viral Diseases, National Center for Immunization and Respiratory Diseases, CDC.

The Global Polio Eradication Initiative (GPEI) continues to make progress toward the eradication target. Only one of the three serotypes, wild poliovirus (WPV) type 1 (WPV1), is still circulating, and the numbers of cases and countries with endemic transmission are at record lows. With the certification of wild poliovirus type 2 (WPV2) eradication in 2015 and the global replacement of trivalent oral poliovirus vaccine (tOPV) containing Sabin poliovirus types 1, 2, and 3 with bivalent OPV containing only Sabin poliovirus types 1 and 3 during April–May 2016, poliovirus type 2 (PV2) is now an eradicated pathogen. However, in eight countries (Cameroon, Chad, Democratic Republic of Congo, Mozambique, Niger, Nigeria, Pakistan, and Syria), monovalent type 2 OPV (mOPV2) was authorized for large-scale outbreak control after tOPV withdrawal (*1*). Poliovirus containment, an evolving area of work that affects every country, aims to ensure that all PV2 specimens are safely contained to minimize the risk for reintroducing the virus into communities. This report summarizes the current status of poliovirus containment and progress since the last report (*2*), and outlines remaining challenges. Within 30 countries, 86 facilities have been designated by the relevant national authorities (usually the Ministry of Health) to become poliovirus-essential facilities for the continued storage or handling of PV2 materials; each country is responsible for ensuring that these facilities meet all biorisk management requirements.

The Polio Eradication and Endgame Strategic Plan 2013–2018 (Endgame Plan) (*3*) of GPEI addresses four objectives: 1) poliovirus detection and interruption; 2) immunization systems strengthening and OPV withdrawal; 3) containment and certification; and 4) transition planning (previously referred to as legacy planning). Under objective 2, the Endgame Plan outlines the readiness criteria and the trigger point for initiating the phased withdrawal of vaccine viruses, starting with Sabin poliovirus type 2. The certification of eradication of WPV2 in 2015 activated the implementation of the containment work.

Indigenous WPV2 was last detected in 1999; it was certified as eradicated in September 2015 by the Global Commission for the Certification of the Eradication of Poliomyelitis (GCC). WPV type 3 (WPV3) was last detected in November 2012 in Nigeria. WPV1 is the only serotype that is endemic and that is in parts only of three countries (Afghanistan, Nigeria, and Pakistan). Four World Health Organization (WHO) regions (Americas, Europe, South-East Asia, and Western Pacific) are certified as polio-free by their respective Regional Certification Commissions (RCCs). Globally, reported WPV1 cases decreased from 74 in 2015 to 37 in 2016; in 2017, six WPV1 cases were reported as of mid-June (*4*).

The predominant risk associated with PV2 after Sabin type 2 withdrawal is the emergence of type 2 circulating vaccine-derived poliovirus (cVDPV2). Since Sabin type 2 withdrawal, GPEI has responded to the emergence or continued transmission of cVDPV2 in Democratic Republic of Congo, Nigeria, Pakistan, and Syria. Large-scale mOPV2 campaigns were conducted in these countries and the Lake Chad basin countries (*5*). Additional PV2 risks include immunodeficient carriers of VDPV (iVDPV), containment breaches by facilities, and deliberate release and “de novo” generation of PV2. To minimize the risks for paralytic poliomyelitis associated with PV2, vaccination with the inactivated poliovirus vaccine (IPV) will be needed for the foreseeable future (*6*).

There are known sources of PV2 in laboratories and vaccine production facilities, and unknown sources, primarily in nonpolio laboratories, including large sample collections of materials collected for other public health or research purposes in areas and at times when PV2 was still circulating. The continuing need for IPV requires the maintenance and expansion of global capacity to produce IPV, which contains PV2 (as well as PV1 and PV3). Because IPV is produced by inactivating wild or attenuated (Sabin) vaccine strains, vaccine production facilities, including quality control laboratories, are a major potential source of live virus. Diagnostic and research laboratories will continue to be needed to ensure rapid diagnostic capacity and critical research on the development of new vaccines and diagnostic methods. In addition, nonpolio laboratories might store and manipulate specimens collected from communities during a period of endemic WPV2 transmission or use of Sabin type 2 vaccine in immunization programs.

The Global Action Plan to Minimize Poliovirus Facility–Associated Risk After Type-Specific Eradication of Wild Polioviruses and Sequential Cessation of Oral Poliovirus Vaccine Use (GAPIII) (*7*), endorsed by the World Health Assembly in 2015, sets the stage for the implementation of containment work. GAPIII, including the annexes, provides the basis for drafting additional guidance documents, and can be revised as new information emerges relevant to achieving the appropriate balance between community risk and the systems and controls to manage that risk (*8*).

GAPIII divides containment work into three phases. Phase I addresses risks of WPV2 and cVDPV2, Sabin type 2 viruses, and potentially infectious materials in polio and nonpolio laboratories with the aim to “destroy, transfer, or contain” PV2 to eliminate or manage the risks. Phase II focuses on implementation of containment in poliovirus-essential facilities designated by countries to serve essential national or global functions, such as vaccine production, diagnostics, or research. Phase III will begin only after eradication of WPV1 and WPV3, and will focus on containment for all PVs.

The global oversight group for containment is GCC (*9*), which determined in 1995 that successful implementation of containment was a prerequisite for global certification. The containment activities in Phase I are overseen by National Certification Committees, which report to RCCs; they, in turn, report to GCC (Figure). Containment activities in Phase II are managed by the National Authorities for Containment (NACs), in consultation with the Containment Working Group, which reports directly to GCC. Technical and scientific issues related to GAPIII are addressed by the Containment Advisory Group. In the past, general containment issues were brought to the Strategic Advisory Group of Experts on Immunization (SAGE), which is the principal advisory group to WHO for vaccines and immunization.

**FIGURE:**
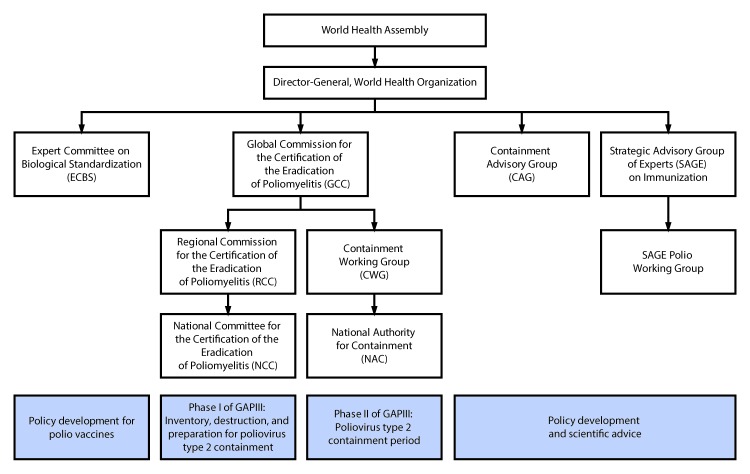
Organizational chart for groups involved in the worldwide containment of poliovirus type 2, including GAPIII* — World Health Organization **Abbreviation: **GAP = Global Action Plan. * WHO Global Action Plan to Minimize Poliovirus Facility–Associated Risk After Type-Specific Eradication of Wild Polioviruses and Sequential Cessation of Oral Poliovirus Vaccine Use. http://polioeradication.org/wp-content/uploads/2016/12/GAPIII_2014.pdf.

The Expert Committee on Biologic Standardization (ECBS) is setting the standards for vaccine production and control to meet the quality standards and requirements for procurement by United Nations agencies. Technical Report Series 926, adopted in 2004, addresses the production and control of IPV in the containment era and is being revised for review and endorsement by ECBS in October 2018. The revised Technical Report Series 926 and GAPIII will be closely aligned. GCC, Containment Advisory Group, ECBS, and SAGE all report directly to the Director-General of WHO; the Director-General, in turn, reports to the World Health Assembly.

## Phase I Progress

The basis for containment work is the national inventory of laboratories and vaccine production facilities that hold and plan to retain PV2 materials. Inventories of PV2 infectious materials were completed in all 194 WHO member countries and 21 territories, and reviewed by RCCs, but might require further scrutiny in some instances (e.g., in countries where mOPV2 has been deployed for outbreak response, inventories will need to be redone after the last campaign round).

All 146 laboratories in the Global Polio Laboratory Network (GPLN) had implemented Phase I activities as of July 31, 2016; as soon as PV2 is detected by these laboratories, the isolates are to be transferred to a poliovirus-essential facility for further processing and sequencing. All original samples and all derivatives with PV2 are to be stored under lock and key when the final results from sequencing become available.

**Wild poliovirus type 2**. Phase I inventories for WPV2 have been completed. Facilities holding WPV2 have been identified, all have implemented the “destroy, transfer, or contain” guidance, and some have been designated as poliovirus-essential facilities (Table).

**TABLE:** Facilities planning to retain poliovirus type 2 (PV2),* by World Health Organization (WHO) region, facility type, and PV2 strain^†^

WHO region	No. of countries	No. of facilities planning to retain PV2 materials	Type of PV2 materials retained and no. of facilities			No. of Salk-IPV production sites	No. of Sabin-IPV production sites^§^	No. of diagnostic or research laboratories
			WPV2	Both WPV2/VDPV2 and OPV2/Sabin2	Only OPV2/Sabin2			
AFR	2	2	0	2	0	0	0	2
AMR	5	27	3	20	4	1	1	25
EMR	2	2	0	0	2	0	1	1
EUR	14	32	5	24	3	8	2	22
SEAR	2	7	1	0	6	0	6	1
WPR	5	16	0	4	12	0	11	5
**Total**	**30**	**86**	**9**	**50**	**27**	**9**	**21**	**56**

**Abbreviations:** AFR = African Region; AMR = Region of the Americas; EMR = Eastern Mediterranean Region; EUR = European Region; IPV = inactivated polio vaccine; OPV = oral poliovirus vaccine; SEAR = South-East Asia Region; VDPV2 = type 2 vaccine–derived poliovirus; WPR = Western Pacific Region; WPV2 = type 2 wild poliovirus.

* Includes WPV2/circulating VDPV2 and OPV2/Sabin2.

^†^ Data as of June 18, 2017.

^§^ Includes potential future producers in different clinical and preclinical phases of Sabin-IPV development.

**cVDPVs and Sabin type 2**. In Nigeria (and contiguous areas around Lake Chad), Democratic Republic of Congo, Pakistan, and Syria, cVDPV2 circulation required extensive use of mOPV2, which was also used in Mozambique after detection of VDPV2 in an area with low vaccination coverage. These detections and ensuing use of mOPV2 have delayed progress toward cVDPV2 and Sabin type 2 containment.

**Potentially infectious materials.** WHO is overseeing the development of guidance documents for potentially infectious materials, which will include a general introduction to containment for nonpolio laboratories, a hazard assessment guide that laboratories can use to determine the risk levels of their materials, and a document that outlines how to raise issues to the Containment Advisory Group. WHO plans to have the guidance documents endorsed by the Containment Advisory Group before the end of 2017.

## Phase II Progress

**Designation of poliovirus-essential facilities and establishment of National Authorities for Containment.** By mid-June 2017, a total of 86 poliovirus-essential facilities had been designated in 30 countries (Table) by government authorities, including 21 (14.4%) of 146 GPLN laboratories. Eighteen of 30 countries with poliovirus-essential facilities had reported establishment of NACs to WHO. NACs, in consultation with the GCC Containment Working Group, will monitor the application process using three levels of certificates: certificate of participation, interim certificate of containment, and certificate of containment (*8*). The final authority for auditing facilities, and issuing these certificates, are NACs.

To support countries with designated poliovirus-essential facilities, WHO has conducted two series of training activities, including GAPIII implementation workshops since February 2015 and containment auditor workshops in 2017; these activities have been attended by 300 participants from all WHO regions.

## Discussion

The scope and complexity of PV containment work are considerable and will affect all 194 WHO member countries and 21 territories for decades to come. Containment of WPV2 is nearing completion, and cVDPV2 and Sabin type 2 containment are in progress, pending the control of cVDPV2 outbreaks. At the same time, issues related to facilities with potentially infectious materials are being addressed.

GPEI expects to achieve eradication of WPV1 in the near future. After global certification of WPV1 and WPV3 eradication, it is anticipated that all Sabin vaccines will be withdrawn, and no new seeding with Sabin viruses should occur. At that point, the world will enter the final stage of containment.

Because PV2 reintroduction into communities could reestablish endemic and epidemic poliovirus transmission, it is critical for this risk to be reduced as close as possible to zero. Countries are aware of this threat and are attempting to decrease the number of facilities handling PV2. The spill of WPV2 in a production facility in the Netherlands in April 2017, infecting one operator, who, in turn, excreted this virus into the public sewage system (documented by environmental surveillance), highlights that the risk for containment breach is not a theoretical risk but something to be anticipated and planned for (*10*). The spill also emphasizes the need for appropriate facility-level biorisk management, incident response planning, and government oversight by NACs.

**Summary**

**What is already known about this topic?**

Poliomyelitis eradication is nearing completion. To sustain eradication, vaccine production, diagnostic, and research facilities retaining polioviruses will have to ensure that these polioviruses are appropriately contained to minimize the risk for release into communities.

**What is added by this report?**

This report summarizes the progress toward implementation of the World Health Organization Global Action Plan for containment (GAPIII), achieved since the declaration of eradication of wild poliovirus type 2 in September 2015 and the withdrawal of Sabin type 2 poliovirus from the trivalent oral poliovirus vaccine in April 2016. Since then, the majority of countries decided not to retain poliovirus type 2, and 30 countries designated 86 poliovirus-essential facilities to address the critical needs for poliovirus vaccine production, disease diagnosis, and research.

**What are the implications for public health practice?**

Effective containment is a prerequisite for the global certification of poliomyelitis eradication. All countries have already compiled inventories of facilities with poliovirus. Countries planning to retain polioviruses are designating poliovirus-essential facilities, establishing national authorities for containment, and are expected to collaborate with the Global Commission for the Certification of the Eradication of Poliomyelitis on global containment oversight.
